# Preliminary Evidence of Improvement in Adolescent and Young Adult Cancer Survivors’ Brain Health Following Physical Activity: A Proof-of-Concept Sub-Study

**DOI:** 10.3233/BPL-210124

**Published:** 2021-10-19

**Authors:** Maude Lambert, Amanda Wurz, Andra M. Smith, Zhuo Fang, Jennifer Brunet

**Affiliations:** a School of Psychology, University of Ottawa, Ottawa, Ontario, Canada; b School of Human Kinetics, University of Ottawa, Ottawa, Ontario, Canada; c Cancer Therapeutic Program, Ottawa Hospital Research Institute, Ottawa, Ontario, Canada; d Institut du savoir Montfort, Hôpital Montfort, Ottawa, Ontario, Canada

**Keywords:** Exercise, neuroimaging, cancer survivors, fractional anisotropy, functional connectivity

## Abstract

**Background:**

Cognitive impairment is common among adolescent and young adult (AYA) cancer survivors. Physical activity (PA) may help mitigate cognitive impairment post-treatment by positively impacting two indicators of general brain health: fractional anisotropy (FA) and functional connectivity (FC). As part of a two-arm, mixed-methods pilot randomized controlled trial (RCT), this sub-study was designed to provide preliminary proof-of-concept evidence for the effects of PA on FA and FC among AYA cancer survivors post-treatment to help inform decisions about proceeding to larger trials.

**Methods:**

AYA cancer survivors who had completed cancer treatment and who were enrolled in a larger pilot RCT comparing a 12-week PA intervention to a waitlist control group, were invited to participate in this sub-study. Sub-study participants completed diffusion tensor imaging and resting-state functional magnetic resonance imaging prior to randomization and post-intervention. Data were analyzed with descriptive statistics, independent component analysis, and paired sample *t*-tests.

**Results:**

Post-intervention, participants showed increases in FA of the bilateral hippocampal cingulum, left anterior corona radiata, middle cingulum, left anterior thalamic radiation, and left cerebellum. A decrease in overall FC of the default mode network and increases in the cerebellar and visual networks were also noted post-intervention (*p* < .05).

**Conclusion:**

Results provide preliminary evidence for the possible positive effects of PA on FA and FC among AYA cancer survivors post-treatment. On the basis of these results, larger trials assessing the effects of PA on specific brain health indicators, as captured by FA and FC, among AYA cancer survivors are appropriate and warranted.

## INTRODUCTION

Cancer-related cognitive impairment (CRCI) is common among adolescent and young adult (AYA) cancer survivors^1^ and can persist post-treatment [1, 2]. Rey et al. [3] found that 58% of AYA cancer survivors have difficulties with memory and/or attention. Additionally, Prasad et al. [4] found that AYA cancer survivors reported problems with organization, memory, emotional regulation, and task efficiency. CRCI, which can impact a wide range of cognitive areas such as executive function, language, and memory [5, 6], can be particularly problematic for AYA cancer survivors who are expected to attain self-sufficiency, have mature relationships, assume more responsibilities, and engage in educational pursuits [1, 7]. Moreover, CRCI can profoundly impact cancer survivors’ health and quality of life, impair psychosocial functioning, and cause disruptions in vocational pursuits, personal relationships, and social and family functions, regardless of age [8–11]. Nevertheless, little has been done to identify interventions to mitigate CRCI among AYA cancer survivors post-treatment.

### Cognitive function and physical activity

There is growing evidence that physical activity (PA) can help to improve cognitive function in older adults and individuals with diseases of cognition such as mild cognitive impairment and Alzheimer’s disease [12, 13]. Evidence also suggests that PA may help to minimize CRCI [see 14 for review]. Gokal et al. [15] found improved self-reported cognitive function in middle-aged breast cancer survivors following a home-based aerobic PA intervention. Similarly, Galiano-Castillo et al. [16] reported improved performance on standardized neuropsychological tests of attention in middle-aged breast cancer survivors following a resistance PA intervention. However, most trials assessing the effect of PA on cognitive function in cancer survivors have focused on middle-aged and older cancer survivors primarily diagnosed with breast cancer [1, 14]. Further, self-reported measures of CRCI have been favored, with the European Organization for Research and Treatment for Cancer Quality of Life Questionnaire [EORTC QLQ; 17] being the most commonly used [14]. Although widely accepted as a valid and reliable measure for cancer-specific quality of life, the EORTC QLQ was not specifically designed to assess CRCI and comprises only two items assessing cognitive function. This may be problematic especially when cognitive symptoms vary widely and are subtle, but clinically meaningful. Whilst studies focusing on survivors’ self-report of CRCI are important as they reflect survivors’ experiences and difficulties in their everyday functioning, it has been suggested that scores might be reflective of mood and psychological distress rather than actual cognitive impairment [18]. Studies examining the association between PA and brain function/structure as objectively assessed via neuroimaging suggest post-treatment reductions in white matter integrity and neuronal activity. Yet, most of these studies are observational and include middle- and older-aged breast cancer survivors [1, 19].

### Neuroimaging methods to investigate the effects of PA on brain function/structure

#### Diffusion tensor imaging

Neuroimaging methods are increasingly being used to investigate the effect of PA on the brain. For instance, diffusion tensor imaging (DTI) can be employed as a non-invasive neuroimaging technique to study white matter architecture (anatomy and structure) in living humans [20]. More specifically, DTI enables the mapping of the diffusion process of water molecules and complex anatomical investigations of different fiber tracts, revealing microscopic details about brain tissue properties [21]. Different measurement parameters can be derived from DTI, including fractional anisotropy (FA), which is a scalar value that provides valuable information about white matter integrity [22, 23]. FA is the most commonly used and studied DTI measurement, and it is a widely accepted measure of white matter integrity due to its high sensitivity to microstructural alterations [21, 24]. Generally, higher FA values are considered a valid index of brain maturity and good health [25].

Although experimental research on the effects of PA using DTI is scarce, some observational studies have reported a positive association between cardiovascular fitness and/or PA and FA in healthy children as well as in younger and older adults from the general population [26–28]. Opel et al. [29] found that better cardiovascular fitness was associated with greater FA in young adults. The authors also found that FA was positively correlated with several cognitive outcomes including processing speed, fluid reasoning, and cognitive flexibility. Similarly, Svatkova et al. [30] and Voss et al. [23] demonstrated that young, middle-aged, and older healthy adults who engaged in an aerobic PA intervention (i.e., biking and walking, respectively) had improved white matter connectivity, that is, increased FA, following the intervention. Collectively, these findings support the notion that PA may have beneficial effects on FA among healthy adults. Considering this, and given that white matter abnormalities have been linked to CRCI in several studies with adult cancer survivors [31–34], examining FA using DTI might be an appropriate and relevant method that can be used to explore the effects of PA on AYA cancer survivors’ brain health post-treatment.

#### Resting-state functional magnetic resonance imaging

Functional magnetic resonance imaging (fMRI) is another method that could be appropriate to offer insight into the effects of PA on AYA cancer survivors’ brain health post-treatment. fMRI is a neuroimaging modality that uses the natural magnetic properties of the body to create detailed images of the brain and measure changes in cerebral blood flow during brain activity [35]. fMRI employed without external stimulation of brain activity (i.e., resting state fMRI [rsfMRI]) allows for the examination of the intrinsic topology of large-scale brain networks and provides information about spontaneous neuronal activity [36, 37]. It provides a measure of functional connectivity (FC), an indicator of brain health referring to the statistical relationship between specific neurophysiological signals of two brain regions in time. Importantly, greater FC indicates a greater degree of co-activation/synchrony (or communication) between brain regions [38]. Yet, decreased global FC within the brain has been reported in children and middle-aged cancer survivors (not specifically AYA cancer survivors) and has been associated with poorer performance in neuropsychological testing [36, 39, 40].

There is experimental evidence to suggest that PA has a beneficial effect on FC within the brain. Burdette et al. [41] found that healthy older adults who were at risk of cognitive decline showed greater resting FC in their hippocampal networks following a 4-month aerobic PA intervention. Additionally, Weng et al. [42] found that engaging in 30 minutes of moderate-intensity aerobic cycling increased resting FC within brain regions associated with affect and reward processing, learning and memory, and attention and executive control in healthy younger and older adults. Thus, experimental studies using rsfMRI could confirm whether PA also has a beneficial effect on FC within the brain of AYA cancer survivors.

### Current study

Given that cancer survivors often exhibit reduced FA and altered FC post-treatment and that PA has beneficial effects on FA and FC in the general population (as reported in studies cited above), exploring whether PA can positively impact these brain health indicators among AYA cancer survivors specifically is required. A necessary first step to explore these associations is to conduct a proof-of-concept (PoC) study. A PoC study could identify whether there is a preliminary efficacy signal of PA on FA and FC in AYA cancer survivors prior to engaging in larger, more comprehensive, and resource-intensive studies. Thus, as part of a two-arm, mixed-methods pilot randomized controlled trial (RCT), the objective of the current PoC sub-study was to assess the effects of a 12-week PA intervention on FA (as measured by DTI) and resting FC (as measured by rsfMRI) in AYA cancer survivors post-treatment. Although it was anticipated that FA and overall FC would improve post-intervention (as compared to pre-intervention), thereby suggesting an overall improvement in the brain health of AYA cancer survivors, no formal hypothesis was put forward given the PoC design of this sub-study.

## METHODS

### Design

This PoC sub-study was part of a larger two-arm, mixed methods pilot RCT study aimed at exploring the effects and feasibility of a 12-week PA intervention on physical and psychological outcomes in AYA cancer survivors. The protocol was registered in the ClinicalTrials.gov database (NCT03016728) and ethical approval was obtained from the Ottawa Health Science Network Research Ethics Board (20160612-01H), the University of Ottawa Research Ethics Board (A03-17-06), the Children’s Hospital of Eastern Ontario Research Ethics Board (16/110X), and the Royal Ottawa Mental Health Centre Research Ethics Board (2017013). Additional details regarding the pilot RCT can be found in Wurz and Brunet [43].

### Participants

AYA cancer survivors in the pilot RCT were recruited through self- and healthcare-provider referrals from different medical clinics and hospitals across a 12-month period, which was specified a priori to assess trial and intervention feasibility and acceptability. Inclusion criteria to participate in the pilot RCT were having: (a) been diagnosed with cancer between the age of 15 and 39 years, (b) completed cancer treatment within the past 5 years with no evidence of progressive or recurrent disease, (c) medical clearance to participate in PA, and (d) the ability to read, understand, and provide informed consent in English. Additionally, AYA cancer survivors had to answer negatively to the question: “*Are you currently engaging in moderate physical activity, which is activity that increases your heart rate and causes you to sweat, more than 3 days/week?*” Exclusion criteria for the pilot RCT were: (a) having a physical impairment preventing engagement in PA, and (b) being unwilling or unable to provide consent.

Once AYA cancer survivors were deemed eligible to participate in the larger pilot RCT, they were given the option to take part in additional assessments including the neuroimaging sessions, which is the focus of the current article. Exclusion criteria for the PoC sub-study described herein included: (a) having metal implants (e.g., pacemaker) or metal dental work (aside from fillings) that would preclude scanning, (b) being claustrophobic/significantly uncomfortable in small spaces, (c) having poor eyesight uncorrectable with contact lenses and precluding stimuli viewing in the scanner, (d) being left-handed (given language lateralization), and (e) being unable to lay relatively still for 1 hour. Additionally, those who answered affirmatively to the following question were excluded: “*Have you been told, in the last 5 years by your healthcare provider that you have a substance use disorder?*”

### Sample size

Power calculations were not performed herein given the reduced statistical power and potential inability to detect statistically significant effects inherent to the small-scaled design of PoC studies [44]. Rather, recruitment remained open and was tracked over a 12-month period to collect feasibility data.

### Procedures

After providing written informed consent and being enrolled into the pilot RCT, all participants completed a comprehensive baseline assessment (week 0) at a location of their choosing (e.g., the University of Ottawa, their home, a local cancer support organization). The assessment included behavioral, physical, and psychological assessments, as well as a qualitative interview. Following the completion of the baseline assessment, participants were informed if they were allocated to the PA intervention group (*n* = 4 for the current PoC study) or the waitlist control group (*n* = 5 for the current PoC study). The second author informed participants of their allocation, which was performed by an independent researcher (who had used a random number generator without a pre-established allocation ratio). Participants allocated to the PA intervention group received a 12-week individualized PA program immediately. Participants in the waitlist control group were asked to continue with their usual routine for 12 weeks, after which point they received a 12-week individualized PA program in the same way as the intervention group. All participants completed a mid-intervention/waiting period assessment (week 6; physical, psychosocial, and behavioral assessments) and a post-intervention/waiting period assessment (week 12; physical, psychosocial, and behavioral assessments, qualitative interview). At study cessation, all participants were entered into a draw to win a $250 CAD gift card.

In addition to the procedures described above, participants enrolled in the current PoC sub-study also completed neuroimaging sessions at the Royal Ottawa Mental Health Centre at three time points: pre-intervention (week 0), post-intervention/waiting period (week 12), and follow-up/post-intervention (week 24). Pre- and post-intervention data were analyzed herein in order to assess DTI and rsfMRI within-person changes following the PA intervention. As such, scans at week 0 were treated as “pre-intervention” data for all participants as this was their first encounter with the scanner, and “post-intervention” data were scans that occurred immediately after receiving the 12-week PA intervention (i.e., week 12 for the intervention group and week 24 for the waitlist control group).

#### The intervention

The 12-week PA program comprised of 4 weekly PA sessions. All participants were given a yoga mat, water bottle, sweat towel, and socks to keep, and were provided with hand weights, resistance bands, and a Polar A300 monitor and heart rate strap, which were returned post-intervention. Two weekly sessions focused on resistance training (e.g., squats, lunges, shoulder presses) performed for 1–3 sets of 6–12 repetitions under the supervision of a certified personal trainer (with cancer-specific training) from weeks 1–6; weeks 7–12 resistance training sessions were unsupervised. The other two weekly sessions focused on aerobic training (e.g., jogging, rowing, walking) performed at 40–75% of participants’ heart rate reserve (self-monitored using a Polar A300 monitor and/or a 10-point Perceived Exertion Scale). These sessions were unsupervised throughout (weeks 1–12). Each PA session lasted between 25–45 minutes, and the volume and intensity of training sessions were individualized for each participant and adjusted based on their progress.

### Data collection

Multiple assessments were completed in the context of the pilot RCT. Only those directly related to the objective of the current PoC sub-study are presented below. Further details regarding the pilot RCT, including assessments, are presented in Wurz and Brunet [43].

#### Socio-demographic and medical information

Prior to randomization, participants self-reported their sex, age, education, work status, age at cancer diagnosis, cancer type, and treatment types. They also reported the frequency and duration of their leisure-time PA (i.e., PA performed during one’s free time) at mild, moderate, and vigorous intensity using a modified version of the Leisure Time Exercise Questionnaire [45]. These data were collected to describe the sample.

#### Neuroimaging sessions

All neuroimaging sessions lasted 1 hour and were performed using a 3 Tesla Siemens Biograph Magnetom MR-PET scanner (Siemens, Erlangen, Germany) equipped with a 12-channel head coil. The DTI sequence included 56 axial slices using a single-shot echo-planar imaging sequence (TR/TE = 10200/80 ms, field of view (FOV) = 256×256 mm^2^, slice thickness = 2 mm, band-width = 1776 Hz/Px). Whole brain echo planar fMRI included 48 axial slices and was performed for the rsfMRI using a gradient echo pulse sequence (TR/TE = 3000/34 ms, FA 90°, FOV = 200×200 mm^2^, voxel size = 1.6 mm×1.6mm×3 mm, slice thickness = 3 mm, band-width =2894 Hz). Sessions also involved task-based fMRI; details are presented elsewhere [46].

### Data processing and imaging analysis

#### DTI analysis

The DTI analysis focused on FA changes from pre- to post-intervention; however, no formal hypothesis testing was undertaken because the current PoC sub-study was underpowered for this. Preprocessing of the DTI images was conducted using the Functional Magnetic Resonance Imaging of the Brain (FMRIB) software Library tool [FSL, version 5.0.9;,47]. For each participant, a brain mask was generated by applying the Brain Extraction Tool-box (BET) to the b0 image. The raw DTI images were then corrected for motion and residual eddy current distortion, and motion parameters were estimated from the transformation matrices for each participant. Appropriate b-matrix rotations were performed in order to account for the rotation component of registration. After motion correction, each participant’s voxel-wise diffusion tensor was calculated using nonlinear estimation [48]. The Tract-Based Spatial Statistics procedure pipeline was used to perform two-sample paired sample *t*-tests to examine FA differences between pre- and post-intervention scans [49]. Whole-brain voxel-wise analysis was conducted at a set threshold of *p*_uncorr_  = 0.05, and a set cluster size larger than 20 voxels.

#### rsfMRI preprocessing

Resting-state images were pre-processed using the CONN toolbox Version 18b [50]. The functional data were functionally realigned and unwarped; slice time corrected; scrubbed with artifact-based identification for outlier scans; segmented into grey matter, white matter, and cerebrospinal fluid, and normalized to the Montreal Neurological Institute template; and smoothed using an 8mm Gaussian kernel. The structural data were segmented into grey matter, white matter, and cerebrospinal fluid, and normalized to the Montreal Neurological Institute template. Artifact detection was run using the ART CONN-toolbox. During the de-noising processes, white matter, cerebrospinal fluid, and outliers detected by the ART toolbox were entered into the linear regression as confounding effects. Subject motion correction was performed via realignment parameters entered in the linear regression of confounding effects. No significant differences between sessions were found for realignment average raw scores (*t*(5) = 0.05, 2-tailed *p* = .48). Linear de-trending was performed, and the default band-pass filter of [0.008 0.09] Hz was applied.

#### Identification of resting-state networks using independent component analysis

Independent component analysis (ICA) is referred to as a “model-/hypothesis-free” method as it allows for the detection of underlying patterns of FC without any pre-defined seed region or a-priori assumptions [51]. Using default settings for CONN 18b, group ICA on the entire dataset from both sessions (pre- and post-intervention) was performed with the FastICA algorithm [52], which is a commonly adopted analytical method for rsfMRI data [53]. Group ICA aims to detect coherently activated brain regions, i.e., independent components (ICs), from all available data and then estimates individual-level ICs to recapture inter-subject variability. As such, it allows to establish direct correspondence of ICs across participants, thereby avoiding the difficulties of matching ICs across individuals [54]. In the current PoC sub-study, computation of the estimated number of detectable networks, i.e., ICs, was performed. The number of independent components to be estimated was set to 40 and dimensionality reduction was set to 64. The correlational spatial match-to-template approach was used in CONN to calculate the correlations between each group-level spatial map (i.e., 40 ICs) and CONN’s default resting network templates to identify the best-matched ICs. The ICs whose correlation coefficients (*r*) are over 0.50 were considered as valid resting state networks, and these ICs were visually inspected and selected for the further group-level analysis. In order to assess the presence of preliminary evidence an effect of PA on FC, paired sample *t*-tests were performed with these ICs between pre- and post-intervention scans at the whole brain level, with the threshold at uncorrected *p* < .01, and false discovery rate (FDR) corrected *p* < .05 at the cluster level.

## RESULTS

### Participants characteristics

Among the 16 AYA cancer survivors who participated in the larger pilot RCT (see Fig. 1 in Wurz et al. [46] for flow diagram), nine were interested, eligible, and consented to participate in the neuroimaging sessions and thus enrolled into this PoC sub-study. After the first session, three withdrew and data from six participants (*n* = 3 intervention group; *n* = 3 wait-list control group) were available for analysis for the current PoC sub-study. Upon visually inspecting the data, one participant was identified as an outlier (based on age at diagnosis, age at study participation, self-reported sex, leisure-time PA) and was subsequently removed from the data set after performing sensitivity testing to ensure similar results with/without the participant. Table 1 summarizes baseline demographic and clinical characteristics of the five participants included in the current PoC sub-study. Briefly, all participants were females and employed. They had undergone surgery for breast cancer (*n* = 3), ovarian cancer (*n* = 1), or soft tissue sarcoma (*n* = 1). On average, they were 37.7 years of age at week 0, diagnosed with cancer at 34.8 years of age, had completed treatment 1.9 years prior to taking part in this sub-study, and reported engaging in 20 minutes (*SD* = 28.3) of moderate-to-vigorous intensity PA per week.

**Fig. 1:**
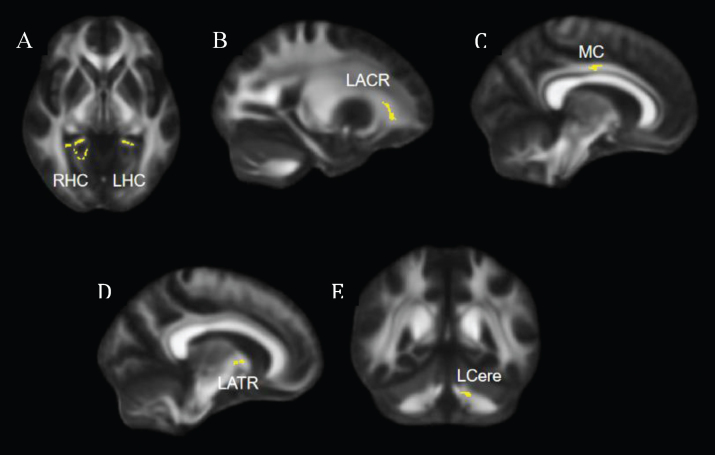
Regions that showed significant increase in FA at post-intervention compared to pre-intervention. (A) Axial view, significant clusters in bilateral hippocampal cingulum. (B, C, D) Sagittal views, significant clusters shown in left anterior corona radiata (B), middle cingulum (C), and left anterior thalamic radiation (D). (E) Coronal view, significant clusters shown in the left cerebellum. FA = fractional anisotropy; LHC = left hippocampal cingulum; RHC = right hippocampal cingulum; LACR = left anterior corona radiata; MC = middle cingulum; LATR = left anterior thalamic radiation; LCere = left cerebellum.

**Table 1:** Characteristics of participants at baseline (*N* = 5)

Characteristics	Values
Demographic
Age [mean years [SD] (range)]	37.7 [2.0]
	(35–40)
Married or living with a life partner [*n* (%)]	4 (80.0)
Highest level of education attained [*n* (%)]
Completed college or undergraduate degree	2 (40.0)
Completed graduate degree	2 (40.0)
Employment [*n* full-time (%)]	4 (80.0)
Clinical
Age at diagnosis [mean years [SD] (range)]	34.8 [2.1]
	(31–37)
Cancer type [*n* (%)]
Breast	3 (60.0)
Soft tissue sarcoma	1 (20.0)
Ovarian	1 (20.0)
Treatment received [*n* (%)]
Surgery	5 (100.0)
Chemotherapy	4 (80.0)
Radiotherapy	3 (60.0)
Hormone therapy	2 (40.0)
Cancer stage [*n* (%)]
Stage III	4 (80.0)
Do not know	1 (20.0)

### Preliminary evidence of the effects of PA on brain health indicators

#### DTI results

Compared to the pre-intervention, participants showed significantly greater FA in the bilateral hippocampal cingulum, left anterior corona radiata, middle cingulum, left anterior thalamic radiation, and left cerebellum post-intervention (Fig. 1).

#### rsfMRI results

**Fig. 2:**
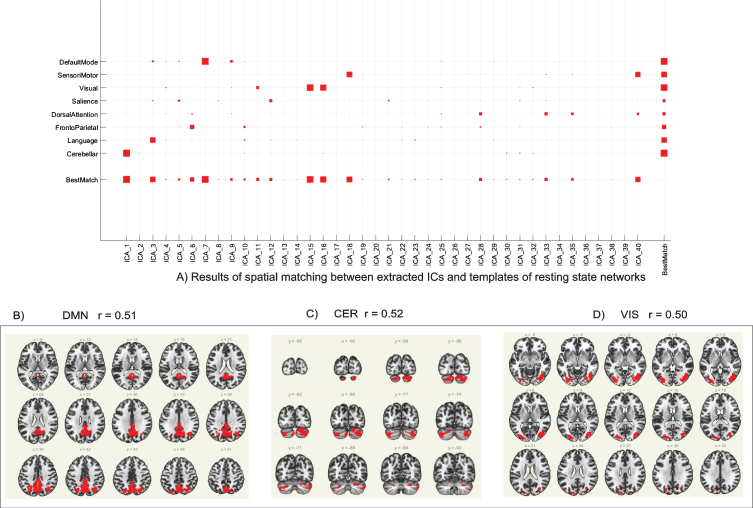
Group ICA results. (A) Results of spatial matching between extracted ICs and templates of resting state networks. Independent components whose correlation coefficient (*r*) > 0.50 were used for further analysis. (B) Group ICA of DMN (ICA7, *r* = 0.51); (C) Group ICA of CER (ICA1, *r* = 0.52). (D) Group ICA of VIS (ICA15, *r* = 0.50). ICA = independent component analysis; IC = independent component; DMN = posterior default mode network; CER = cerebellar network; VIS = visual network.

As previously described in the **Methods** section, the group ICA was performed on the entire dataset (pre- and post-intervention scans for all participants) to detect underlying patterns/networks of FC (i.e., ICs) without any pre-defined seed region or a-priori assumptions (Fig. 2A). ICs whose correlation coefficient was *r*>0.50 were used for further analysis, which includes the posterior default mode network (DMN; ICA7, *r* = 0.51; Fig. 2B), the cerebellar network (CER; ICA1, *r* = 0.52; Fig. 2C), and the visual network (VIS; ICA15, *r* = 0.50; Fig. 2D).

**Fig. 3:**
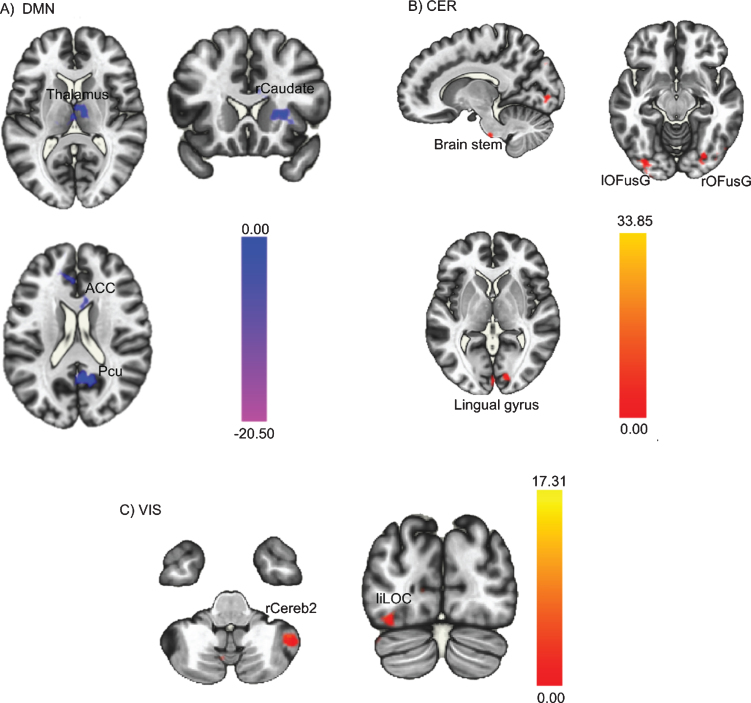
Whole-brain level paired sample *t*-test results of FC differences between pre- and post-intervention. Red regions indicated increased FC after the PA-intervention and blue regions indicated decreased FC after the PA-intervention. (A) Decreased DMN FC; (B) Increased CER FC; (C) Increased VIS FC. The threshold was set as uncorrected *p* < 0.01 at the whole brain level, and reported regions survived at *p_*FDR*_* < 0.05 at the cluster level. FC = functional connectivity; PA = physical activity; DMN = posterior default mode network; CER = cerebellar network; VIS = visual network.

Results of paired sample *t*-tests, performed to assess FC changes in the three identified ICs from pre- to post-intervention, show that FC decreased in the DMN, increased in the cerebellar network, and increased in the visual network post-intervention compared to pre-intervention. This is depicted in Fig. 3, whereby the colour red indicates an increase in FC post-intervention compared to pre-intervention and the colour blue indicates a decrease in FC. Specifically, Fig. 3A illustrates the decrease in the FC of the DMN, including regions within the DMN such as the anterior cingulate cortex and precuneus, and also a few regions outside the DMN such as the thalamus and the right caudate. Figure 3B shows the increase in the FC of cerebellar network post-intervention, more specifically, the FC between cerebellar network and brain stem, bilateral occipital fusiform gyrus, and right lingual gyrus. Finally, Fig. 3C illustrates the increase in the visual network, including the right cerebellum Crus2 and left inferior lateral occipital cortex. Data on specific regions and detailed *t*-tests statistics are reported in Table 2.

**Table 2:** Differences in FC between pre- and post-intervention scans

Network	Region	Voxels	Peak MNI coordinates			*t*-value	*P*_FDR_
			x	y	z		Cluster-level
*Post-intervention* < *pre-intervention*
DMN	ACC	47	–18	42	20	16.06	0.02
	Pcu	207	2	–56	20	9.71	0.003
	Thalamus	126	–2	–18	10	20.5	< 0.001
	Right caudate	33	22	20	4	16.67	< 0.001
*Post-intervention* > *pre-intervention*
CER	Brain stem	38	10	–12	–42	10.96	0.045
	Left OFusG	97	–38	–84	–12	16.51	< 0.001
	Right OFusC	85	24	–82	–14	26.44	0.001
	Right Lingual gyrus	47	12	–88	2	13.59	0.001
VIS	Left iLOC	116	–32	–82	–10	13.84	0.008
	Right CerebII	119	46	–52	–44	13.8	0.042

FC = function connectivity; MNI = Montreal Neurological Institute; DMN = default mode network; ACC = anterior cingulate cortex; Pcu = precuneus; OFusG = orbital fusiform gyrus; CER = cerebellar network; VIS = visual network; iLOC = inferior lateral occipital cortex; CerebII = cerebellum cortical Crus II.

Given the importance of cognitive resting networks to the present PoC sub-study, we visually inspected the remaining ICs, compared them to the network templates, and applied a liberal correlation coefficient (*r* > 0.40) to explore the cognitive resting networks. Two ICs were identified to be matched with the language network (*r* = 0.41) and sensorimotor network (*r* = 0.43). The detailed results are reported in supplementary material.

## DISCUSSION

Cancer survivors often exhibit reduced FA and altered FC post-treatment [39, 40, 55], although no studies examining this among AYA cancer survivors specifically have been done. The aim of the current PoC sub-study was to detect preliminary evidence on the role that PA might play in promoting FA and FC within the brain among AYA cancer survivors to make informed decisions on whether to proceed with larger, more expensive trials. More specifically, using DTI and rsfMRI methods, the objective of the current PoC sub-study was to assess the effects of a 12-week PA intervention on FA and FC within the brain among AYA cancer survivors post-treatment.

The DTI results indicated post-intervention increases in FA of the bilateral hippocampal cingulum, left anterior corona radiata, middle cingulum, left anterior thalamic radiation, and left cerebellum. Moreover, the rsfMRI results demonstrated a post-intervention decrease in the overall resting FC of the DMN and post-intervention increases in the cerebellar and visual networks. Together, these results indicate a favorable preliminary efficacy signal, meaning that PA may have positive effects on AYA cancer survivors’ brain function/structure. On the basis of these results, continued investigation into the effects of PA on FA and FC among AYA cancer survivors is warranted. If effects are confirmed, it could inform the development of PA interventions aimed at promoting brain health among AYA cancer survivors.

Although it is important to consider that these findings have limited immediate implications given the small-scaled design of the current PoC sub-study, they offer preliminary evidence that PA may positively impact FA of different brain regions in AYA cancer survivors and provide an essential foundation for future studies. If replicated in larger trials, results would suggest that PA may drive an adaptive response positively promoting the reversal/restoration of some of the cancer-related white matter alterations. Whilst PA may impact FA through various mechanisms, one mechanism that may be important to consider when further investigating the effects of PA on FA is the brain derived neurotropic factor, which is produced by muscles and in brain tissues during PA and acts as a key regulator of neuroregeneration, synaptogenesis, as well as synaptic plasticity (Rasmussen et al., 2009; Seifert et al., 2009). As such, future studies investigating the PA-FA relationship in AYA cancer survivors should consider the neurotropic and neuroprotective functions of the brain derived neurotropic factor within this association [56–58].

Additionally, the current PoC sub-study also offers preliminary evidence that PA may positively impact FC in AYA cancer survivors post-treatment. Sufficiently-powered studies will be critical to confirm these associations. The post-intervention decrease in FC of the DMN and increases in FC of the cerebellar and visual networks observed herein exemplify the expected antagonist relationships between “task-negative” networks (i.e., networks that exhibit higher levels of activity when resting in a quiet state with no external stimulation) –such as the DMN –and “task-positive” networks (i.e., networks that exhibit higher levels of activity when engaging in goal-oriented mental activity requiring attention) –such as the cerebellar and visual networks [59, 60]. Within the wider literature, it has been established that the activation of these two types of networks needs to be asynchronous for optimal brain health and cognitive performance [61, 62]. Although only preliminary, the results of the current PoC sub-study are compelling and warrant further exploration in larger trials. If replicated in future studies, it would suggest a PA-induced adaptive cognitive process, making the brain more “calm” in resting state and thereby more ready to engage in cognitive tasks when required [63].

### Study strengths and limitations

To our knowledge, despite its small-scaled design, this PoC sub-study represents the first effort to combine both DTI and rsfMRI methods to examine the brain of AYA cancer survivors before and after a PA intervention. Given the substantial incidence of CRCI among cancer survivors and the lack of robust evidence-based treatment strategies to reduce its burden and severity, early stage research (including PoC studies) aiming to explore the role of PA in counteracting/managing CRCI is warranted and the consideration of FA and FC as contributors to these cognitive deficits might be important. Studies involving neuroimaging data are even more essential given their paucity in the wider literature and AYA cancer survivor literature specifically [14]. Nevertheless, the results of the current PoC sub-study should be interpreted in light of important limitations. First, the results are based on a small, convenience sample of AYA cancer survivors who identified as female and were mostly employed. Second, this PoC sub-study did not include long-term follow-up assessments nor comparisons between the intervention and the waitlist control groups. Third, although participants were instructed to lie calm and not to think of anything during the rsfMRI scanning sessions, compliance to these requests could not be monitored and brain activity interference could therefore not be entirely excluded. Fourth, it is important to consider that the favorable FA and FC changes observed herein might not be driven by the PA intervention solely but also by the byproducts of the intervention. For example, PA generally leads to improvements in both psychological and physical health and well-being [64, 65]. Accordingly, factors such as decrease in body mass index, increase in cardiovascular fitness, and improved mental health may act as confounding variables in the observed results [66]. These limitations may have implications on the generalizability of the findings and suggest further research exploring the role of PA on brain health in male and unemployed AYA cancer survivors is warranted. It is also necessary to consider the role of potential confounding factors including demographic and medical variables in future trials. Finally, testing the underlying mechanisms involved in the PA-FA/FC relationships and long-term effects of PA on FC and FA is needed to increase our understanding of *how* PA impacts AYA cancer survivors’ brain health.

## CONCLUSION

The present PoC sub-study offers support for the continued investigation into the beneficial effects of PA on the brain among AYA cancer survivors post-treatment. Although the implications and generalizability of the current results are limited due to the small-scaled design of PoC studies and have to be interpreted cautiously, the fact that significant statistical effects were observed in such a small sample size is promising and suggests further testing of the impact of PA on brain health using DTI and rsfMRI following cancer treatment is justified and warranted.

## Footnotes

^1^
In line with the National Cancer Institutes’ definition, cancer survivors are defined herein as individuals from the time of diagnosis until the end of life.

## TRIAL REGISTRATION

clinicaltrials.gov, NCT03016728. Registered January 11, 2017, clinicaltrials.gov/ct2/show/NCT03016728.
